# A model for the effects of germanium on silica biomineralization in choanoflagellates

**DOI:** 10.1098/rsif.2016.0485

**Published:** 2016-09

**Authors:** Alan O. Marron, Helen Chappell, Sarah Ratcliffe, Raymond E. Goldstein

**Affiliations:** 1 Department of Applied Mathematics and Theoretical Physics, Centre for Mathematical Sciences, University of Cambridge, Wilberforce Road, Cambridge CB3 0WA, UK; 2 Medical Research Council Human Nutrition Research, Elsie Widdowson Laboratory, 120 Fulbourn Road, Cambridge CB1 9NL, UK; 3 School of Biochemistry, Biomedical Sciences Building, University of Bristol, University Walk, Bristol BS8 1TD, UK

**Keywords:** germanium, silicon, biomineralization, toxicity, first principles modelling, choanoflagellate

## Abstract

Silica biomineralization is a widespread phenomenon of major biotechnological interest. Modifying biosilica with substances like germanium (Ge) can confer useful new properties, although exposure to high levels of Ge disrupts normal biosilicification. No clear mechanism explains why this disruption occurs. Here, we study the effect of Ge on loricate choanoflagellates, a group of protists that construct a species-specific extracellular lorica from multiple siliceous costal strips. High Ge exposures were toxic, whereas lower Ge exposures produced cells with incomplete or absent loricae. These effects can be ameliorated by restoring the germanium : silicon ratio, as observed in other biosilicifying organisms. We developed simulations of how Ge interacts with polymerizing silica. In our models, Ge is readily incorporated at the ends of silica forming from silicic acid condensation, but this prevents further silica polymerization. Our ‘Ge-capping’ model is supported by observations from loricate choanoflagellates. Ge exposure terminates costal strip synthesis and lorica formation, resulting in disruption to cytokinesis and fatal build-up of silicic acid. Applying the Ge-capping model to other siliceous organisms explains the general toxicity of Ge and identifies potential protective responses in metalloid uptake and sensing. This can improve the design of new silica biomaterials, and further our understanding of silicon metabolism.

## 1. Introduction

Silicon (Si) biomineralization is the formation of biological structures from amorphous hydrated silicon dioxide (silica). Biosilica structures have various functions, from support and protection [1] to light wavelength modification [2] and detoxification [3]. Biosilicification is a widespread phenomenon, occurring in diverse animals, plants and protists. These include ecologically important groups (e.g. grasses, diatoms), making biosilicification a major component process of global biogeochemical cycles [4]. Biosilicification also has biomedical relevance, with evidence for a role for silicon in connective tissue and bone formation [5].

A major aspect of biosilica research regards its potential biotechnological applications, including as drug delivery vehicles, biosensors, catalytic systems and tissue engineering scaffolds [6,7]. Biomineralized silica is formed at significantly milder conditions of temperature and pH compared with artificial silica glasses [7] and biosilicifying organisms produce precisely nanopatterned biosilica structures. These highly replicable patterns are species-specific due to the strict genetic controls involved in their formation [8]. Furthermore, biosilica structures have enhanced strength, due to the hierarchical organic–inorganic microstructure, to the extent that diatom frustules are the strongest biological materials per unit density known to science [1].

Biosilica can also gain new properties via surface modifications [9]. Doping biosilica with substances such as germanium (Ge), boron (B) and titanium (Ti) confers new functions such as photoluminescence, electroluminescence and photocatalysis [9–12]. Incorporation of these substances occurs at low levels under natural conditions [13], but they can have major consequences for biosilicification at higher exposure levels.

Uptake and accumulation of germanium into biosilica structures have been documented in sponges [14,15], chrysophytes [16,17] and diatoms [18–20]. Germanium is toxic to rice plants [21], inhibits growth and scale formation in chrysophytes [16,17], coccolith formation in some haptophytes [22] and causes spicule deformities in sponges [14,15]. Diatoms have an optimum ratio for Ge incorporation into the frustule biosilica before the toxicity of Ge exposure affects biosilicification [23]. Exposing diatoms to Ge at low levels results in frustule deformities, while at higher Ge levels the disruption to diatom Si metabolism and inhibition of the cell cycle is lethal to the organism [19].

Multiple studies have established that Ge toxicity is not dependent on absolute dosage, but rather the Ge : Si ratio that organisms are exposed to [24]. However, no clear mechanism exists as to why Ge disrupts biosilicification, beyond the assumption that it is connected to the similarities of Ge and Si at the atomic level [25]. Molecular mimicry explains how Ge is transported by heterologously expressed Si transporters from vertebrates [26], plants [27] and diatoms [28], and yet this shows that Ge is not intrinsically harmful to cells. Instead, Ge toxicity must be connected to specific Si-related processes, as it has no effect on non-siliceous organisms [22].

**Figure 1.**
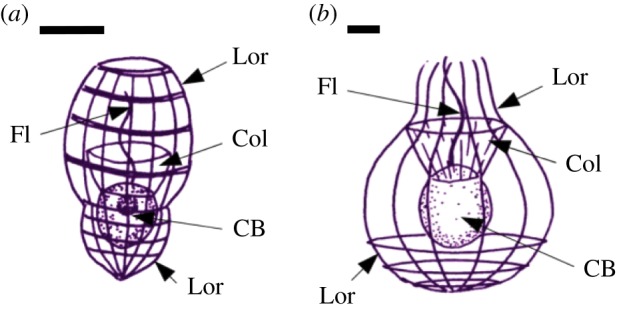
The loricate choanoflagellates *Stephanoeca diplocostata* (*a*) and *Diaphanoeca grandis* (*b*). All choanoflagellates have the same basic form consisting of an ovoid cell body (CB) with a single apical flagella (Fl) surrounded by a collar (Col) made of microvilli. Loricate choanoflagellates are characterized by their extracellular siliceous basket, the lorica (Lor), whose size and shape is species-specific. Scale bar, 5 µm. Schematics are based on [30] and illustrations from the Micro*scope 6.0 website (http://starcentral.mbl.edu/microscope, drawings by Won-Je Lee).

One major biosilicifying lineage is the loricate choanoflagellates (Acanthoecidae), which are members of a heterotrophic protist group closely related to animals and fungi [29]. Loricate choanoflagellate cells are characterized by the possession of a siliceous extracellular basket, or lorica (figure 1). The lorica is constructed from a series of siliceous rods (costal strips), and lorica morphology is species-specific [29]. Silicification begins with silicic acid uptake and concentration within intracellular silicon deposition vesicles (SDVs) [30]. Silica polymerization then proceeds within the SDV, induced by organic components [31] and shaped by microtubule-mediated expansion of the SDV [29]. Each SDV produces an individual costal strip that is then exocytosed to the cell surface in a characteristic sequence [29].

In the nudiform condition, costal strips are produced de novo and directly assembled into a new lorica. In the tectiform condition, costal strips are stored on the cell surface at the collar region (figure 1) until cell division. The daughter cell inherits a full set of strips and assembles a lorica immediately, before commencing strip production for the next generation. While the nudiform and tectiform conditions are generally characteristic of different species [29], there exists some flexibility in the timing of lorica construction. *Stephanoeca diplocostata* is normally tectiform in culture, but silicon-starved cells will eventually cease costal strip production. These cultures will continue to be aloricate until there is sufficient silica to resume costal strip production, at which point cells will first construct their own lorica (i.e. nudiform), before beginning production of costal strips for the next generation (i.e. tectiform). This is in contrast with diatoms, which cease cell division entirely when silicon starved [29]. Choanoflagellates possess silicon transporter (SIT) proteins [30], like those of diatoms and haptophytes. Heterologously expressed SITs exhibit Ge transport capacity and SIT-containing organisms show sensitivity to Ge [22]. It is therefore interesting to study the effect of Ge on loricate choanoflagellates: it would be predicted that their SITs would facilitate Ge uptake, but that the cells themselves could decouple silicification from their wider metabolism in response, and thus be unaffected by Ge.

Here we describe the effects of Ge exposure on two species of loricate choanoflagellate, *S. diplocostata* and *Diaphanoeca grandis* (figure 1). Unexpectedly, Ge was found to be toxic, and increasing the Ge : Si ratio reduced growth rates and disrupted lorica formation. Using observations from these experiments, combined with first principles modelling, we propose a mechanism for the effect of Ge(OH)_4_ on loricate choanoflagellate biology and on biosilicification in general.

## 2. Material and methods

### 2.1. Culture conditions

Cultures of *S. diplocostata*, *D. grandis* and *Salpingoeca rosetta* were grown in sterilized artificial seawater (ASW) (36.5 g l^−1^ Marin Salts (Dr. Biener Aquarientechnik, Wartenberg Germany) in ddH_2_O). Media Si concentrations were measured by silicomolybdate assay [32,33].

Organic enrichment (4 g l^−1^ proteose peptone (Sigma), 0.8 g l^−1^ yeast extract (Fluka Biochemika), autoclaved sterile) was added to the cultures to provide a food source for prey bacteria: 15 µl ml^−1^ ASW for *S. rosetta*, 7.5 µl ml^−1^ ASW for *D. grandis* and *S. diplocostata*. *Salpingoeca rosetta* cultures were grown at 22°C and split every 4–7 days and *D. grandis* and *S. diplocostata* cultures were grown at 13.5°C and split every four to eight weeks.

Two millimolars of stock solutions were made up in 50 ml polypropylene conical tubes (Fisher Scientific) from GeO_2_ (Aldrich), Na_2_SiO_3_ · 9H_2_O (Aldrich) or NaCl (Fisher Scientific) in ddH_2_O. The solubility of GeO_2_ in water (4.4.7 g l^−1^ at 25°C; equal to 42.7 mM) means that Ge is expected to be completely released within a 2 mM solution.

Cells were concentrated for experimental cultures by centrifugation (2700*g* at 4°C for 45 min). Five millilitres of concentrated culture was added to 5 ml ASW and 40 µl organic enrichment in a 15 ml polypropylene conical tube (Fisher Scientific). All +Ge exposures in this study are given as a percentage of 10 ml of 150 µM Si ASW. Experimental +Ge exposures were measured to produce a Ge concentration between 1 and 20% of this Si content. Negative control cultures had ddH_2_O added in a volume equal to the largest volume of Ge(OH)_4_ used for the experimental cultures. For Si-rescue experiments, 3 ml of 2 mM Si(OH)_4_ was added as a percentage (400%) of the 150 µM Si level of 10 ml ASW. Blank controls were established by adding 2 mM NaCl solution at equivalent volumes and time points to the addition of Si(OH)_4_ solution, to verify that results are Si-related, rather than being due to salinity or dilution effects. Cultures were grown at 13.5°C and monitored every 48 h.

### 2.2. Microscopy

Morphological observations were carried out using a Zeiss Observer.Z1 microscope at 100× magnification, under DIC and phase contrast. Photographs were taken using a Photron FastCam SA3 and PFV FastCam Viewer v. 333 (Photron Ltd, 2006). Cell body lengths along the long axis were measured with the Fiji software package [34] and statistically analysed using independent sample Student's *t*-tests. Cell counts were performed with a Neubauer Improved 0.100 mm haemocytometer using a Nikon TE2000-U microscope at 20× magnification, under bright field. Cell concentrations were calculated from the mean of three replicate counts. Each experiment involving cell counts was repeated separately three times from fresh starting cultures. The results of the cell counts at each time point were statistically analysed with a nested mixed-effects general linear model run through SPSS v. 24 (IBM, USA). Where a significant effect of Ge treatment was observed at a time point, a Tukey's HSD *post hoc* comparison was performed to determine which Ge conditions were significantly different.

±Ge cultures of *D. grandis* were fluorescent stained using Lysotracker Red DND-99 (Life Technologies). The larger *D. grandis* lorica was preferable for observing changes in cell morphology and costal strip accumulations. Two millilitres of concentrated culture was added to 2 ml ASW and 16 µl organic enrichment. Ge(OH)_4_ was added as a calculated percentage of Si, as before, with ddH_2_O used as a negative control. Seven hundred and fifty nanomolars of Lysotracker Red DND-99 was added to the cultures from a 500 µM stock solution prepared by dilution in sterile ASW. Experimental cultures were kept at 13.5°C in the dark for 12 days. Confocal fluorescence microscopy imaging was performed at 100× magnification using Zeiss Axiovert 200M inverted microscope, LSM 510 laser system (Jena, Germany) and the Zen2010 software (Zeiss).

### 2.3. First principles modelling

Geometry optimization of substituted silica structures were carried out using the plane-wave density functional theory (DFT) code, CASTEP [35]. The generalized gradient approximation (GGA) and PBE exchange-correlation functional were employed [36], along with on-the-fly pseudo-potentials for greatest accuracy. Convergence testing determined a kinetic cut-off energy of 650 eV and sampling of the Brillouin zone was carried out at the γ point [37] in a simulation box of *a* = *b* = *c* = 25 Å and *α* = *β* = *γ* = 90°. The simulation box size was determined from convergence testing to ensure repeat images did not interact through the periodic boundary conditions. The simulation box size remained fixed throughout the simulations. Convergence tolerances for energy change, maximum displacement and maximum force were set at 1 × 10^−5^ eV per atom, 0.001 Å, and 0.03 eV Å^−1^, respectively.

A starting model of Si_8_O_16_ was constructed as a representative, small, amorphous silica structure. This was taken from a paper [38] reporting *ab initio* structures of the formula (SiO_2_)*_N_* up to an *N* value of 12. Substitutions of Ge for Si were made in this structure at all Si positions in turn. A completely Ge-substituted model was also constructed. Without substitution, this starting structure was also modelled with the addition of a single GeH_3_OH molecule, placed freely in the simulation cell away from the main structure. Formation energies were typically calculated as shown in equation (2.1).2.1
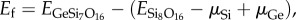
where 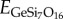 is the energy of a singly Ge-substituted silica molecule, 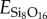
the energy of a single silica molecule, 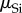 the chemical potential of silicon and 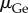 the chemical potential of germanium. All formation energies were calculated in the same way, using identical conditions and chemical potentials, to ensure robust comparison. The chemical potentials for Si and Ge were calculated from monoclinic silicon and bulk Ge (space group Fd-3 m), respectively.

## 3. Results and discussion

### 3.1. Effect of germanium on choanoflagellate morphology and growth rates

**Figure 2.**
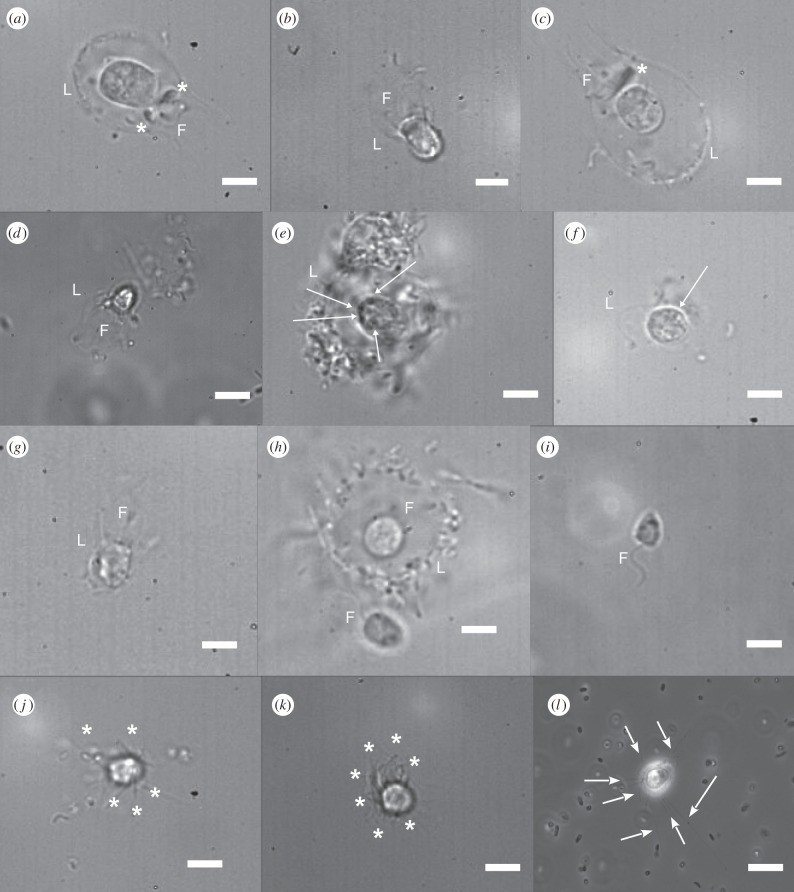
Ge exposure produces aberrant morphologies in loricate choanoflagellates. (*a*) *Diaphanoeca grandis* cell grown in +Ge ASW. (*b*) *Stephanoeca diplocostata* cell grown in +Ge ASW. Both have enlarged cell bodies compared to normal *D. grandis* (*c*) and *S. diplocostata* cells (*d*) from control (no Ge added) cultures. Panels (*e*,*f*) are cells from +Ge cultures, showing necrotic *D. grandis* and *S. diplocostata* cells respectively, both lacking flagella and having cytoplasmic occlusions (arrows). (*g*) *S. diplocostata* cell from a +Ge culture with a partial lorica. The strips around the collar region are present but lightly silicified, and the posterior strips are missing. Panels (*h*) (*D. grandis*) and (*i*) (*S. diplocostata*) are examples of completely aloricate cells from +Ge cultures. The aloricate cell in (*h*) is beside a loricate cell, demonstrating that the size and shape of the cell body is otherwise unaffected. Both (*h*,*i*) have clearly visible flagella and normal collar regions. (*j*,*k*) Juvenile *D. grandis* and *S. diplocostata* cells respectively from −Ge control cultures. These are recently divided cells photographed in the process of lorica assembly. The costal strips are being moved into position and the flagella is absent. (*l*) An aloricate *D. grandis* cell from a −Ge control culture. These cells are encountered infrequently in normal culture conditions. Note the lack of a flagella or collar, and the presence of long, irregularly shaped filopodia (arrows). Within photographs: L, lorica, F, flagella, * costal strips in bundles (*a*,*c*) or in assembly (*j*,*k*). Photographs were taken at 100× magnification under phase contrast. Scale bars, 5 µm.

Ge exposure had marked effects on loricate choanoflagellate morphology. In +Ge cultures, protoplasts were generally enlarged (figure 2*a*,*b*), often accompanied by accumulations of costal strips on the collar (arrow, figure 2*b*). Mean cell body length was 5.8 µm long (±0.2 s.e., *N* = 30) in *S. diplocostata* +3%Ge cultures after 7 days, significantly (*p* < 0.001) larger than in control cultures (e.g. figure 2*c*) where mean cell body length was calculated at 4.9 µm (±0.1 s.e., *N* = 48). *Diaphanoeca grandis* cells grown at 3%Ge were larger (mean = 7.3 µm ± 0.3 s.e., *N* = 13) compared with cells from control cultures (e.g. figure 2*d*) where the mean cell body length was calculated at 6.8 µm (±0.2 s.e., *N* = 27), although this difference was only significant at a 90% confidence level (*p* = 0.087). Necrotic cells were frequently observed at higher Ge concentrations, as evidenced by disrupted cell membranes, absence of flagellar motion and opaque occlusions within the cytoplasm (figure 2*e*,*f*). No morphological effects were seen in +Ge cultures of the non-loricate, non-silicifying species *S. rosetta*.

At lower Ge levels (1–3%), disrupted loricae, including aloricate cells, were commonly observed in both species (figure 2*f*–*i*), but distorted lorica morphologies were rarely seen in control (−Ge) cultures. This was observed after only 2 days, equivalent to the cell division time of *S. diplocostata* [29]. No consistent distortion pattern (i.e. loss of strips, changes in strip size) was recognizable. Aloricate cells were otherwise intact, possessing a normal cell body, collar and flagella (figure 2*h*,*i*), but without costal strip accumulations or lorica-constructing tentacles [29]. This indicates that aloricate individuals were not simply juvenile cells observed before construction of a new lorica (cf. figure 2*j*,*k*). Nor were they the result of recent lorica loss by mechanical means, as aloricate cells were not associated with a nearby empty lorica. Aloricate cells displayed normal flagellar beating, demonstrating that the cells were alive and healthy despite lacking a lorica (electronic supplementary material, video S1). Both loricate and aloricate cells were found in the same +Ge cultures (figure 2*h*).

**Figure 3.**
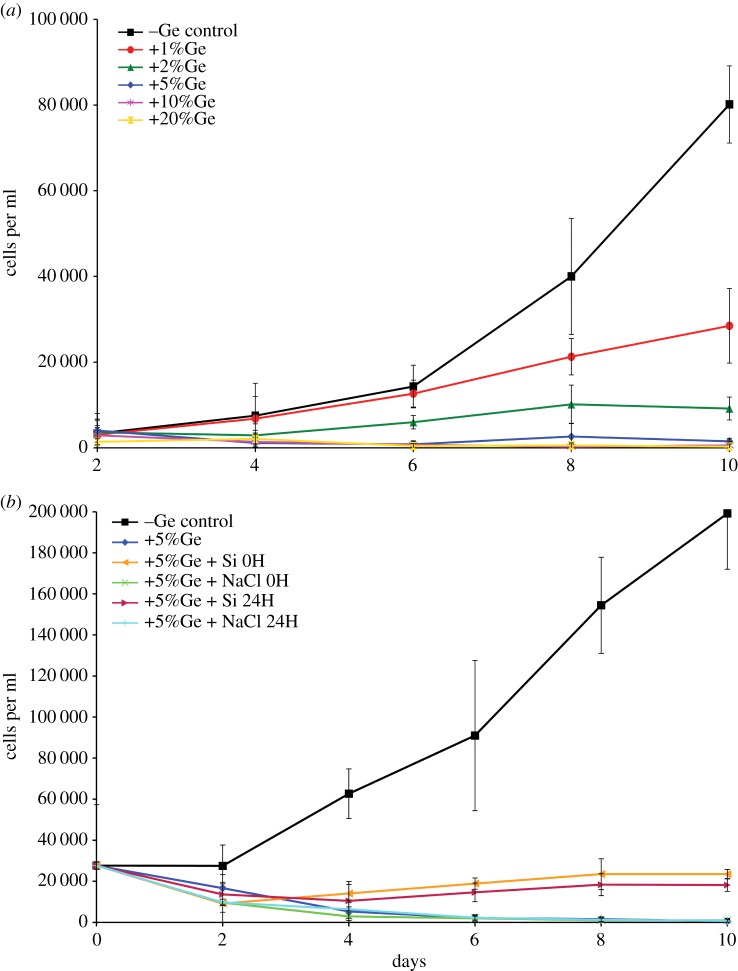
The effects of altering the Ge : Si ratio on growth rates in *S. diplocostata*. (*a*) Haemocytometer cell counts from cultures grown under control (−Ge) up to +20%Ge exposures. The −Ge control shows a normal pattern of exponential increase of cell concentrations over time during the course of the experiment. Exposure to progressively higher levels of Ge results in significantly (*p* < 0.05) lower cell concentrations after 10 days, with exposures of more than 5%Ge being toxic after 10 days. (*b*) Cell counts showing that adjusting the Ge : Si ratio from +5 to +1% can rescue culture growth rates to some degree. Cultures with Si added at set-up (0H) or after 1 day (24H) have significantly lower growth rates than the −Ge control cultures after 4 days (*p* < 0.001). However, the +Si adjusted cultures have significantly higher cell concentrations compared with +5%Ge (positive control) cultures at the end of the 10-day course of the experiment, or to those cultures with a blank (NaCl) rescue solution added at the start (0H) or after 1 day (24H) (*p* < 0.05 in all cases). Error bars are ±s.d. of the cell count measurements calculated from the replicate cultures. See electronic supplementary material, table S1*a*,*c* for statistical analyses.

Ge exposure markedly reduced *S. diplocostata* growth rates (figure 3*a*; see electronic supplementary material, table S1*a* for statistical analyses). Control (−Ge) cultures showed an exponential pattern of growth (doubling time ∼41.5 h). At 1%Ge, cell division rates proceeded roughly similar to control cultures (doubling time = 48.4 h) until 6 days, after which these cultures had slower growth compared with the controls (estimated doubling time approx. 113.7 h). By the end of the 10-day course of the experiments the culture cell concentrations were significantly different from the −Ge control cultures (*p* < 0.01). At 2%Ge, initial growth rates were close to zero, after 4 days division rates increased slowly and after 8 days plateaued. At the end of the 10-day experiment, the cell concentrations of +2%Ge cultures were significantly different to cell concentrations in both lower and higher Ge exposures (*p* < 0.05). Higher Ge exposures had a more pronounced effect on cell division. 5%Ge culture cell concentrations were extremely low for the full 10 days of the experiment (significantly different from controls at *p* < 0.01), and Ge concentrations above 5% were essentially lethal after 4 days. Higher Ge exposures (10 and 20%) also showed significantly lower cell concentrations by the end of the experiment (*p* < 0.05). This corresponds with morphological observations, where at low Ge concentrations new cells were produced with deformed or absent loricae (figure 2*g*–*i*), while at higher %Ge exposures cells were swollen or necrotic (figure 2*a*,*b*,*e*,*f*). By contrast, *S. rosetta* cultures grown at 20%Ge had no significant change in growth rates or cell concentrations compared with −Ge controls (electronic supplementary material, figure S1; see electronic supplementary material, table S1*b* for statistical analyses).

In diatoms and haptophytes, the ratio of Ge : Si is the biologically relevant factor and the impact of increased Ge concentrations is ameliorated by increasing Si concentrations [19,22]. We investigated if this was also the case for loricate choanoflagellates by establishing +5%Ge *S. diplocostata* cultures and then adding Si to adjust the Ge : Si ratio to +1%Ge. This adjusted Ge : Si ratio should, according to Ge-exposure growth curves (figure 3*a*), allow cell division to proceed, whereas blank control cultures (where NaCl is added as the counter ion mix) would be expected to show low or zero growth rates. The Si adjustment was done either at the initial set-up or after a period of time (24 h) to establish if cultures could have their growth rates ‘rescued’.

The results of this experiment are shown in figure 3*b* (see electronic supplementary material, table S1c for statistical analyses). In all cases, +Ge cultures had significantly lower cell concentrations than −Ge control cultures after 4 days (*p* < 0.001). However, addition of 400%Si resulted in significantly higher cell concentrations after 8 days (*p* < 0.01) compared with unadjusted or+NaCl blank control cultures, demonstrating a rescue effect of Si. The outcome was the same (i.e. non-significant *p*-values for +5%Ge+Si0H versus +5%Ge+Si24H at each time point) whether Si was added to the initial culture or after 24 h (figure 3*b*; electronic supplementary material, table S1*c*); however, addition of Si at time points beyond 24 h did not rescue growth (data not shown). This corresponds to *S. diplocostata* cells dividing approximately every 48 h, as culture cell cycles could not be synchronized (cf. diatoms [39]) but would be predicted to have all undergone interphase at some point after 24 h. Costal strips are produced during interphase and, therefore, all cells would have commenced the silicification pathway within 24 h. The time limit for Si-rescue points to a link between Ge and costal strip formation in *S. diplocostata*. After a certain time spent producing costal strips in a high-Ge environment, the cell is irreparably damaged. Interestingly, addition of 400%Si should have effectively restored the Ge : Si ratio to that of the 1%Ge cultures, but the growth curves in figure 3*b* are qualitatively different from the 1%Ge growth curve in figure 3*a* even for cultures where the Ge : Si ratio was restored at initial set-up. This suggests that additional factors related to seawater chemistry may influence Ge toxicity.

The sensitivity of loricate choanoflagellates to relatively low Ge : Si ratios is unexpected. The absence of any Ge effect in non-siliceous choanoflagellates and the dependence on the Ge : Si ratio within the first 24 h indicate that the toxic effect is linked to biosilicification and costal strip formation, rather than to lorica assembly or cytokinesis. In contrast, with diatoms, where silicification is a vital part of the life cycle, exposure to 5%Ge is lethal [19,33]. Loricate choanoflagellates are facultatively siliceous and can survive in zero-silicon media [29]. The modular nature of the lorica and the flexibility in the timing of its construction suggests that biosilicification is a non-essential aspect of choanoflagellate physiology. However, our results demonstrate that Ge is lethal to choanoflagellates at levels tolerated by other organisms such as sponges and barley [14,15,40]. Choanoflagellate Ge toxicity instead resembles that of chrysophytes [16,17], which also are facultatively siliceous and possess multiple siliceous elements produced continually throughout the cell cycle.

### 3.2. Mechanism of the effect of germanium on biosilicification

The general mechanism of metalloid toxicity involves molecular mimicry, due to the similar atomic radii and reactive properties of the semi-metals. Toxins (e.g. arsenite) that closely resemble metabolites (e.g. phosphate) are toxic due to their acting as catalytic poisons through competitive but irreversible binding to enzyme active sites [25]. Such competitive interactions explain Ge transport by SIT proteins [27,28]. Under very low Si conditions, where there would otherwise be insufficient bound Si to permit transport, the resemblance of Si(OH)_4_ and Ge(OH)_4_ allows dissolved Ge to bind to the SIT proteins through allosteric mechanisms, and thus increase SIT uptake activity [33].

Given this molecular biomimicry, there is, to date, no precise mechanism explaining why Ge is toxic to biosilicification. While Ge competes with Si during transmembrane transport, there is no evidence that this is harmful or that it permanently prevents any Si uptake. The effects of Ge must be related to the general process of silica polymerization rather than to specific biosilicification proteins, because Ge toxicity occurs across diverse silicifying organisms. It has been proposed that Ge disturbs the mechanism of oligosilicate stabilization for intracellular transport and, therefore, induces the uncontrolled formation of toxic silica particles within the cytoplasm [19]. Alternative explanations have centred on the assumption that Ge atoms can insert into biosilica [24], somehow disrupting the SiO_2_ structure [23].

**Figure 4.**
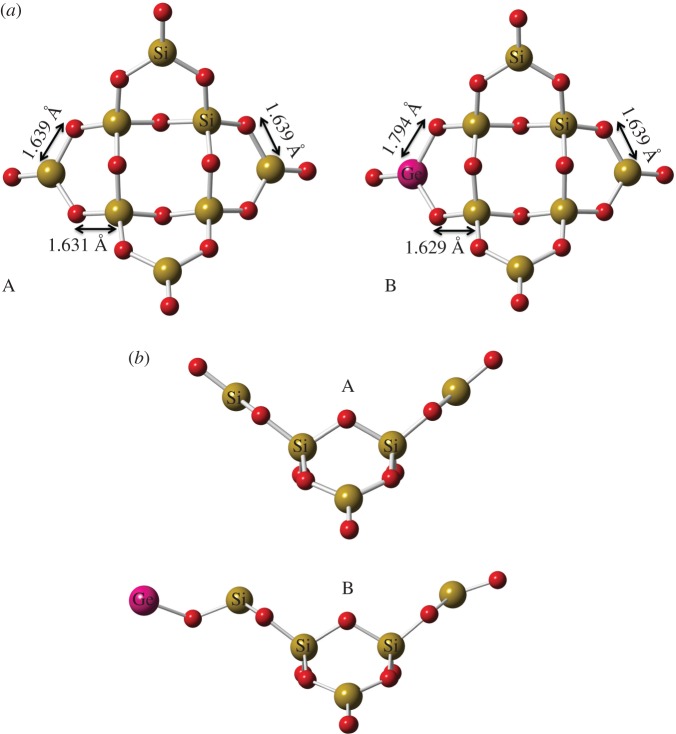
First principles simulations of Ge substitutions in the silica structure. (*a*) The expansion of the silica structure can be seen with a Ge ion substituted into a terminal structural position (B). The bond lengths show that the changes to the structure are local in nature, with more distant bonds being unchanged between the unsubstituted (A) and substituted (B) structures. (*b*) GeH_3_OH was placed freely in the simulation box with the silica unit, and Ge substitution was found to occur at a terminal position (B). The resulting water molecule and two H ions have been removed for clarity, leaving only the Ge substitution. The changes to the structure from the unsubstituted (A) to the substituted (B) are now more pronounced. Yellow, silicon; pink, germanium; red, oxygen.

We, therefore, conducted first principles DFT simulations to examine what happens to the structure of amorphous silica when Ge enters the system, either as a direct substitution for Si or as an additional ion (figure 4*a*). The polymerizing silica was represented as a Si_8_O_16_ structure, which has been shown to be the lowest energy structure for this sized system [38]. When Ge atoms were substituted for Si, the formation energy, which determines the favourability/stability of a substitution, was positive (average 2.8 eV for terminal positions, average 3.7 eV for internal positions). This shows that Ge substitution for Si is energetically unfavourable, but is slightly more favourable in the terminal positions (figure 4*a*). Furthermore, greater levels of Ge substitution were highly energetically unfavourable (up to 26.2 eV to form Ge_8_O_16_). However, the Ge–O bond length in the singly substituted silica structures was only an average of 7.7% greater (7.5–7.7% greater in terminal positions, 7.8–7.9% in internal positions) than the bond length of Si–O. This structural change was local and only marginally changed bond lengths elsewhere in the simulated silica structure. Even a simulated Ge_8_O_16_ structure was merely expanded, rather than fractured by the large concentration of Ge, as might have been expected.

A repeated simulation modelled the addition of Ge as GeH_3_OH, and thus examining the interaction between germanic acid and polymerizing silica. Here, Ge atoms bonded to the end of the silica structure (figure 4*b*), with the formation of a water molecule. The formation energy in this scenario was negative (−2.5 eV), indicating a favourable, stable substitution. Structurally the addition of Ge to the end of the molecule changed the structure, producing a ‘kink’ at either end (figure 4*b*).

The hypothesis that emerges is that Ge (as germanic acid) will readily incorporate onto the ends of a polymerizing silica structure. This would create stable, Ge-capped silica at the external termini with unaffected poly-SiO_2_ in the internal structure. The high formation energy required to grow the silica structure further onto the ends of the Ge-caps means that silicification would stop, resulting in stunted segments. The higher the Ge : Si ratio the more frequent these ‘capping’ events would be, explaining why the Ge : Si ratio is the relevant factor rather than the absolute Ge concentration being critical. This method could be used to optimize the doping of biosilica with other substances (boron, titanium, etc*.*) [10,12].

This hypothesized mechanism of Ge toxicity in siliceous organisms would also depend on the number of individual biosilicification events occurring within cells and the size of the polymerization front. Multiple, individual biosilicification events occurring inside the cell will involve multiple Ge-SiO_2_ interactions, and smaller SiO_2_-polymerization fronts will be more prone due to the limited availability of sites for addition of Si or Ge. Loricate choanoflagellates provide an ideal system to test these aspects of the Ge-capping toxicity model.

### 3.3. Evidence from loricate choanoflagellates for the Ge-capping model

**Figure 5.**
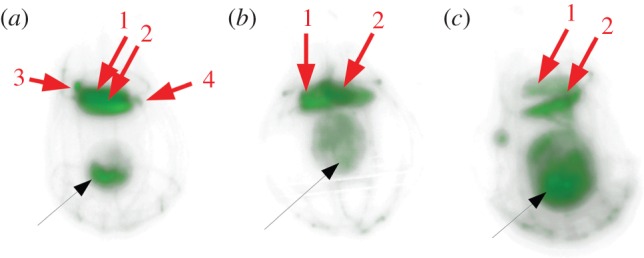
The effects of Ge exposure on *Diaphanoeca grandis* cells. (*a*) Confocal image of a control cell, (*b*) confocal image of a cell grown at +1%Ge and (*c*) confocal image of a cell grown at +2.5%Ge. All cells were incubated with Lysotracker Red DND-99, a stain which fluoresces at acidic pH, and which accumulates and fluoresces within the SDV before eventually becoming incorporated into fluorescent biosilica structures. All cells were imaged under the same conditions of illumination and gain, and images were processed identically. These images show the costal strip bundles (numbered red arrows), demonstrating that +Ge cells have two bundles but only cells in control cultures were observed to possess four bundles. Mature cells accumulate four costal strip bundles just before division. These confocal images also show the degree of fluorescence present in the cell body (black arrow). Control cells have low overall fluorescence, mainly concentrated in compartments at the posterior of the cell. Cells from +1%Ge cultures have a greater amount of fluorescence, in compartments but also present as a diffuse fluorescence throughout the cytoplasm. Cells grown at 2.5%Ge exposure have a large amount of fluorescence throughout the cell body, which is swollen and contains multiple, highly fluorescent compartments. This is evidence for Ge interfering with the process of biosilicification, preventing silica polymerization and leading to a build-up of silicic acid in the cell and within the cytoplasm.

The Ge-capping model is supported by confocal microscopy of Lysotracker-stained *D. grandis* grown at various Ge levels. Lysotracker stain fluoresces at low pH and accumulates in acidic subcellular compartments, including SDVs. Here, it becomes incorporated into polymerizing silica, resulting in fluorescent biosilica structures [41]. In control cultures (figure 5*a*, electronic supplementary material, stack images 1A), the *D. grandis* lorica fluoresced, as did subcellular compartments at the posterior and around the edges of the cell body. These are interpreted as being either food vacuoles or SDVs with forming costal strips [29]. Cells in control cultures had variable costal strip accumulations on the top of the collar. Some cells had four accumulations, indicating a full complement of strips and a cell about to undergo cytokinesis. Others had two accumulations, while some had no strips at all on the collar. This shows that cells in control cultures were at different stages of costal strip production within interphase.

Fluorescent costal strips in +Ge *D. grandis* cultures indicated that some silica polymerization and lorica assembly had occurred. However, many loricae had non-fluorescent strips (i.e. formed before the experiment started) or were incomplete. The protoplast staining pattern of +Ge choanoflagellates was notably different to that of the controls (figure 5, electronic supplementary material, stack images 1). At low Ge (1%), fluorescent subcellular compartments were visible, together with a diffuse staining throughout the cytoplasm (figure 5*b*). At higher Ge levels (3%), the protoplast was larger and both cytoplasmic and subcellular compartment fluorescence was more intense (figure 5*c*). This staining pattern suggests that the cell swelling is due to the build-up of a low pH substance in the cytoplasm and that this increases at higher Ge levels. In +Ge cultures, the majority of cells have accumulations of fluorescent costal strips on the collar, but consistently as two bundles (figure 5*b*,*c*). No cells were ever observed with a mature complement of four bundles. We interpret this as evidence that costal strip production can only proceed to a limited extent, but that the cell cycle and cell division is being retarded by Ge exposure. This is exemplified by electronic supplementary material, figure S2 and stack image 2, showing a necrotic cell with two costal strip accumulations, where stunted strips are mid-way through exocytosis.

Silicifying costal strips are particularly vulnerable to Ge-capping as each strip has only two areas of growth, at either end of the lengthways-expanding SDV [29]. The Ge-capped polymerization fronts would be unable to support further silicification, stunting costal strip growth. Despite biosilicification ceasing, Si(OH)_4_ (and Ge(OH)_4_) import would continue throughout interphase (cf. diatoms [39]). With the lorica size and shape being species-specific, costal strip (and therefore SDV) numbers must also be determinate, so the choanoflagellate cell cannot produce additional SDVs to compensate for disrupted biosilicification. The imported Si(OH)_4_, therefore, builds up in the cell, lowering cytoplasmic pH and swelling the cell, as observed in the light and confocal microscopy (figures 2*a*,*b* and 5*c*). Eventually, Si(OH)_4_ concentrations may become high enough for silica autopolymerization, resulting in highly damaging silica occlusions free in the cytoplasm (figure 2*e*,*f*). Therefore, the choanoflagellate cells will die once they are unable to complete costal strip formation, remove excess silicic acid or divide.

At low Ge exposure (1–3%), costal strip formation is restricted by infrequent Ge-capping events but can still continue, albeit in fewer cells and at a slower rate than under control Ge : Si ratios (figure 3*b*). In many cases, division proceeds with stunted costal strips or with incomplete sets of strips, producing distorted lorica morphologies (figure 2*g*–*i*). This results in slower growth rates and increased mortality as cells cope with moderate Si(OH)_4_ build-up and the energetic costs of disrupted lorica formation.

Low Ge exposures also result in more aloricate cells. We hypothesize that as infrequent Ge-capping slows costal strip formation the choanoflagellate cell has sufficient time to sense the disruption to biosilicification before a lethal Si(OH)_4_ build-up occurs. This sensing system must be endogenous to the cell rather than being controlled by extracellular conditions (because sufficient Si is present for costal strip formation), and is unlikely to be transporter-based because SITs exhibit low discrimination between Ge and Si. The feedback system may be conducted via cytoskeleton–SDV interactions [29] indicating perturbations in the formation and growth of SDVs, and modulating gene expression and Si uptake. Such a cytoskeletal feedback system could be how choanoflagellate cells sense that sufficient strips are present for lorica construction and that it is, therefore, appropriate to undergo cytokinesis. Tectiform species show additional modes of costal strip manipulation and lorica construction, mediated by the cytoskeleton, and a related feedback system may have been involved in the proposed evolution of the tectiform mode of lorica construction and cell division from a nudiform ancestor [29].

In this way, low Ge : Si exposure ratios elicit the same responses in loricate choanoflagellates as growth in zero-Si conditions [29]. Instead of upregulating Si(OH)_4_ import (as in diatoms [33,39]) the *S. diplocostata* cell shuts down costal strip production and switches to the nudiform mode of life. Persisting in the nudiform lifestyle presumably continues until conditions are suitable for costal strip formation to resume. Only some cells experience a suitable Ge : Si uptake ratio or are at a suitable stage in lorica formation to allow switching to the nudiform condition; hence, why 1%Ge cultures are not all aloricate, and why growth rates are still reduced under low Ge exposures. The aloricate cells are unlikely to be mutant lineages that do not biosilicify; if this were the case then similar numbers of live aloricate cells would also be found under all Ge exposures (figure 3).

### 3.4. Wider biological relevance of the Ge-capping model

Applying the Ge-capping model to biosilicification in general explains why the Ge : Si ratio is critical for toxicity. Disruption to biosilicification depends on the frequency of capping events, which in turn depends on the probability that Ge will be imported into the SDV. This holds on the assumption that all Si uptake systems also transport Ge(OH)_4_ across the membrane (valid for all known Si transporters), and is independent of the various biosilica formation mechanisms [8,22,31]. If Ge toxicity was due solely to destabilization of oligosilicate complexes [19,20], then it would be predicted that toxicity would increase with absolute Ge and Si concentrations as this would lead to higher rates of Si(OH)_4_ and Ge(OH)_4_ uptake into the cytoplasm.

For diatoms, disruption at low Ge levels will stunt biosilicification and produce aberrant structures, as observed in *Synedra acus*, *Pinnularia* sp., *Nitzschia alba* and *Thalassiosira pseudonana* [11,19,20,23,24]. At higher Ge : Si ratios, widespread Ge-capping causes frustule growth to cease entirely, preventing cell division and causing cell death [19]. As with choanoflagellates, stunting silica polymerization while continuing to take up Si(OH)_4_ leads to Si(OH)_4_ build-up in the cytoplasm and eventually uncontrolled silica autopolymerization. This phenomenon may have led to observations of cytoplasmic silica granules in *S. acus* cells exposed to Ge [20]. Diatoms display a negatively chemotactic response to Ge-loaded beads [42], which is presumably required to protect the vital biosilicification processes against Ge toxicity. Indeed, it may be that the relative rates of Si polymerization versus Ge-capping forms the basis for this diatom Ge-evading response.

Chrysophytes, haptophytes and loricate choanoflagellates show many common features relating to biosilicification. These groups are unicellular, use SIT-based Si uptake systems and can be facultatively siliceous. They all produce multiple siliceous (or partially siliceous) elements in a species-specific pattern throughout interphase [16,17,22,29,30] . Chrysophytes form non-siliceous cells at low Ge : Si ratios, and exhibit lower growth rates and higher mortality in high Ge conditions [16,17]. Similarly, haptophytes exposed to low Ge levels produce aberrant biomineralized structures, while at higher Ge : Si ratios growth rates are impacted. Despite this, Ge exposure produces no negative effects on the wider metabolism, such as photosynthesis [22]. We, therefore, predict that our model for the effect of Ge exposure would also apply to organisms such as *Synura petersenii*, *Paraphysomonas vestita*, *Scyphosphaera apstenii* and *Coccolithus braarudi*. This highlights how the Ge-capping model can be applied to all biosilicifying organisms across diverse taxonomic groups.

In organisms producing macroscopic silica structures, such as sponge spicules, Ge effects will be most evident at the smallest silicification growth fronts (e.g. spicule tips) where sites become Ge-capped sooner. Growth toward the middle of the spicule can proceed even at high Ge : Si ratios due to the larger silicification front providing enough sites for continued Si polymerization. This would produce stunted spicules with bulbous central regions, as observed by Simpson and co-workers [14,15]. Plants also show Ge toxicity at sites of silicification in the leaves [40]. These effects only occur at very high Ge exposures, with plants largely protected at the point of natural soil Ge exposure. The basis for this protection is proposed to be via Ge discrimination at the point of xylem loading, with Si being able to form organosilicon complexes for xylem transport, while Ge complex formation is energetically unfavourable [43].

Protection by discrimination against Ge may also occur via diatom [33] and sponge [44] active silicic acid uptake, thereby influencing global Ge and Si geochemical cycling. The two-step bioreactor method for incorporating Ge into diatom biosilica [45] overcomes this, and the toxic effects of Ge, by taking advantage of diatom surge uptake after Si starvation [33], and the larger silicification fronts provided by almost-complete thecae [46]. The capping model predicts that Ge incorporation will be at the biosilica surface, unless alternative silica polymerization sites can grow over the terminated front. However, in that case the Ge will be merely coated over by SiO_2_ rather than being truly incorporated into a composite biosilica structure. The Ge-capping model also predicts how the frustule will be affected by the timing of Ge addition; the structures being formed upon Ge exposure will be truncated, and this will be observed most readily in microstructures produced by smaller silicification fronts [19,23,24,45]. This illustrates how understanding the molecular mechanisms of biosilica formation can inform the design and modification of biomaterials and their use in new biotechnological applications [10,11,47].

The apparently universal applicability of Ge-capping can be used to investigate the wider role of silicon in biology [4], even in apparently non-silicifying organisms or cryptic silicifiers [22]. For example, Ge causes cell division anomalies in *Fucus vesiculosus* embryos, despite this species having no known requirement for Si [48]. Interestingly, Ge also exerts toxic effects on mammalian kidneys [49]. While the nature of Si-related physiology in mammals remains incompletely understood [50], a system of Si-transporter proteins in the kidney has been demonstrated [26], and silicon deprivation causes deformities of the bones and connective tissues [5]. The fact that these organs are affected by high Ge : Si ratios further points towards Si–O–Si bonds playing an important part in mammalian, and therefore human, biology.

## Supplementary Material

Supplementary Legends Figures and Table

## Supplementary Material

Supplementary Stack Image 1A

## Supplementary Material

Supplementary Stack Image 1B

## Supplementary Material

Supplementary Stack Image 1C

## Supplementary Material

ESM_Supplementary_Stack_Image_2
