# Supplementary material for: A model for the effects of germanium on silica biomineralization in choanoflagellates
Source: J R Soc Interface. 2016 Sep;13(122):20160485. doi: 10.1098/rsif.2016.0485 (PMC5046948; doi:10.1098/rsif.2016.0485)
Supplement: Supplementary Legends Figures and Table [file rsif20160485supp1.pdf]

Supplementary Figure 1. Altering the Ge:Si ratio of the culture medium has no effect on the growth of *Salpingoeca rosetta*. Measurements are taken from haemocytometer cell counts from cultures grown at either control (-Ge) ASW or +20%Ge, corresponding to the highest Ge exposures used for *Stephanoeca diplocostata* experiments (Figure 3a). Cell count measurements were taken over 6 days due to the higher growth rate of *S. rosetta* compared to *S. diplocostata*, and to avoid possible complications for cell counts as the choanoflagellates switched between swimming, colonial and thecate morphotypes. All cell concentrations were calculated from the mean of triplicate counts on three replicate cultures. Error bars are ± standard deviation of the cell count measurements calculated from the replicate cultures. See Supplementary Table 1b for statistical analyses confirming that there was no significant effect of Ge exposure.

Supplementary Figure 2. Still image of the *D. grandis* cell corresponding to the confocal section image sequence in Supplementary Stack Image 2. Image taken at 100x magnification using phase contrast. Note the enlarged and swollen cell body and high amounts of bacteria (black arrows) inside the lorica, indicating an unhealthy cell. The mottled appearance of the cell body is characteristic of necrotic cells in +Ge cultures. Lor= lorica, Col=collar, CB= cell body, Fl=flagella, *= costal strips in bundles. Scale Bar= 5µm.

Supplementary Stack Image 1. 3D confocal images of a *D. grandis* cell grown under -Ge conditions (A), +1%Ge exposure (B) and +2.5%Ge exposure (C). These stacks correspond to the cells in Figure 4a, b and c respectively. All cells were incubated with Lysotracker Red DND-99, a stain which fluoresces at acidic pH, and which accumulates and fluoresces within the SDV before eventually becoming incorporated into fluorescent biosilica structures. All cells were imaged under the same conditions of illumination and gain, and images were processed identically. X axis= Y axis (1 pixel= 0.15µm), Z axis 1 pixel= 0.9µm.

Supplementary Stack Image 2. 3D Confocal image of a necrotic *D. grandis* cell grown at +2.5%Ge treatment. This cell was cultured with Lysotracker Red DND-99, a stain which fluoresces at acidic pH and which accumulates within the SDV and is incorporated into fluorescent biosilica structures. The costal strips of the lorica are partially fluorescent and two bundles of accumulated costal strips are visible on the collar. Within the cell multiple long fluorescent structures at the margins of the cell body, corresponding to forming costal strips within SDVs. These SDVs are at different stages, as indicated by the different sizes, and the cell has become necrotic due to the inability to correctly regulate and manage these biosilicification processes. X axis= Y axis (1 pixel= 0.15µm), Z axis 1 pixel= 0.9µm.

Supplementary Video 1. An aloricate *Stephanoeca diplocostata* cell demonstrating beating flagella. The initial part of the video moves the focal depth through the z-axis of the cell to show that no lorica or costal strips are present. The presence of a flagella proves that this aloricate cell is a feeding adult rather than it being observed before completion of lorica construction. The flagellar motion indicates that the cell is alive and otherwise normal, apart from the absence of a lorica. Recording taken at 100x magnification using phase contrast. Video recorded at 60fps. Scale bar= 5µm.

Supplementary Figure 1

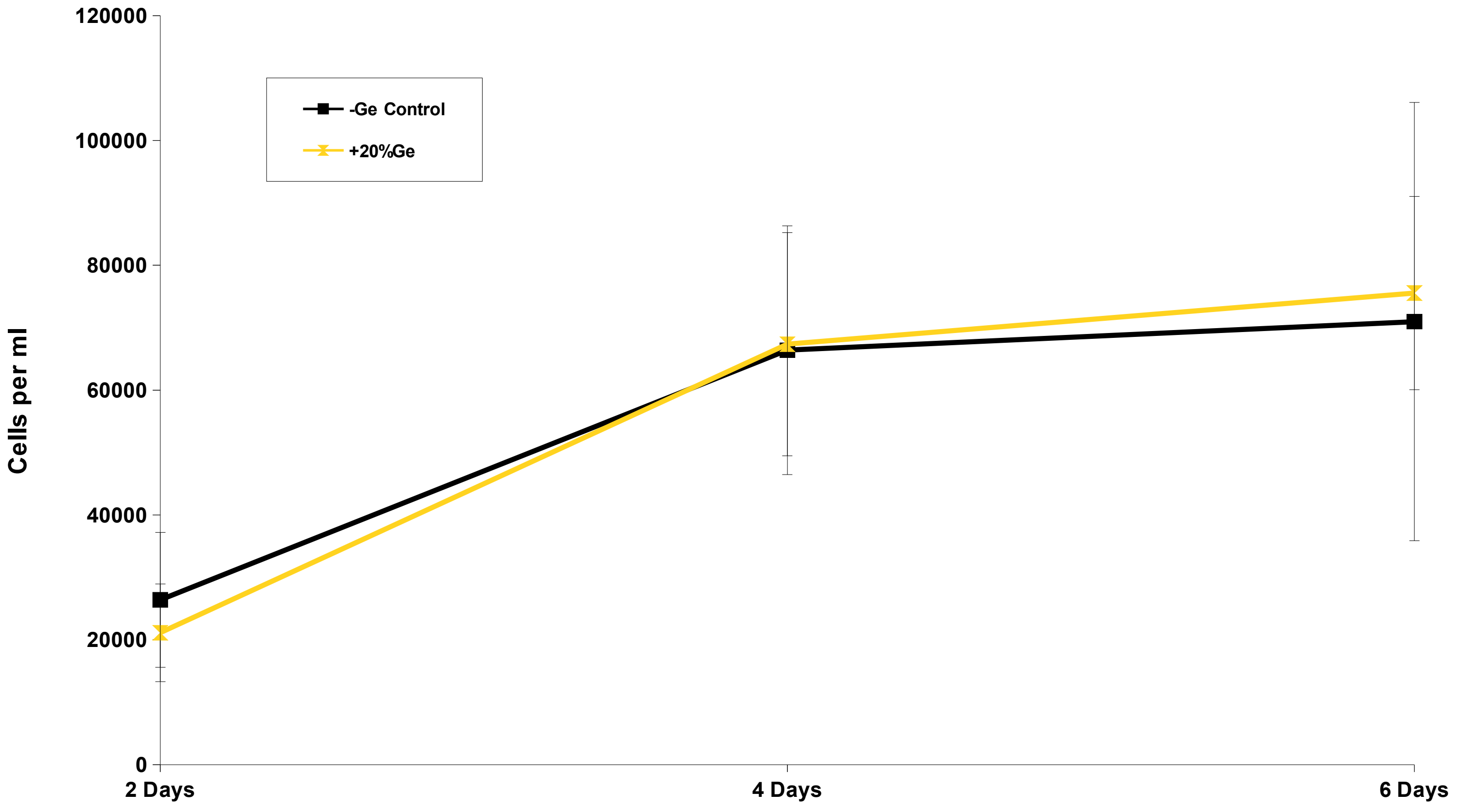

Supplementary Figure 2

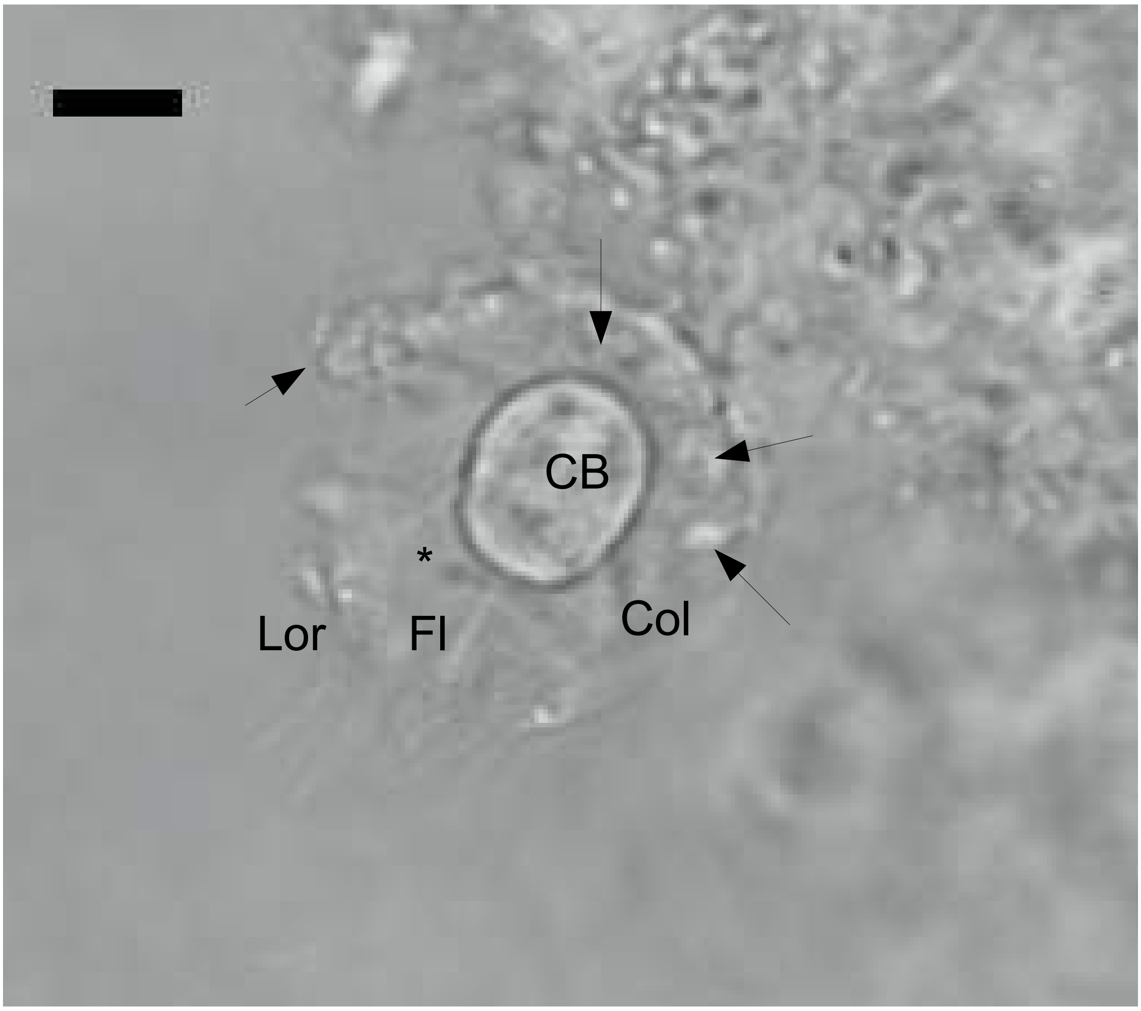

(a)

2 Day Comparison for Figure 3a

Tests of Between-Subjects Effects

Dependent Variable: 2 Day Cell Concentration

**Source**		**Type III Sum of Squares**	**df**	**Mean Square**	**F**	***p***
Intercept	Hypothesis	504166666.700	1	504166666.700	88.898	0.000
	Error	68055555.560	12	5671296.296[a]		
Ge Exposure Treatment	Hypothesis	37152777.780	5	7430555.556	1.310	0.323
	Error	68055555.560	12	5671296.296[a]		
Culture Replicate(Ge Exposure Treatment)	Hypothesis	68055555.560	12	5671296.296	0.778	0.669
	Error	262500000.000	36	7291666.667[b]		

[a] MS(Culture Replicate(Ge Exposure Treatment))

[b] MS(Error)

4 Day Comparison for Figure 3a

Tests of Between-Subjects Effects

Dependent Variable: 4 Day Cell Concentration

**Source**		**Type III Sum of Squares**	**df**	**Mean Square**	**F**	***p***
Intercept	Hypothesis	713223379.600	1	713223379.600	93.367	0.000
	Error	91666666.670	12	7638888.889[a]		
Ge Exposure Treatment	Hypothesis	353964120.400	5	70792824.070	9.267	**0.001**
	Error	91666666.670	12	7638888.889[a]		
Culture Replicate(Ge Exposure Treatment)	Hypothesis	91666666.670	12	7638888.889	0.443	0.934
	Error	620833333.300	36	17245370.370[b]		

[a] MS(Culture Replicate(Ge Exposure Treatment))

[b] MS(Error)

***p*-values from Tukey's HSD *post-hoc* test**	**-Ge Control**	**+1%Ge**	**+2%Ge**	**+5% Ge**	**+10%Ge**
**-Ge Control**					
**+1%Ge**	0.999				
**+2%Ge**	0.205	0.37			
**+5% Ge**	0.027	0.063	0.938		
**+10%Ge**	0.038	0.087	0.969	1	
**+20%Ge**	0.087	0.179	0.998	0.996	0.999

6 Day Comparison for Figure 3a

Tests of Between-Subjects Effects

Dependent Variable: 6 Day Cell Concentration

Source		Type III Sum of Squares	df	Mean Square	F	*p*
Intercept	Hypothesis	1822945602.000	1	1822945602.000	15.181	0.002
	Error	1440972222.000	12	120081018.500[a]		
Ge Exposure Treatment	Hypothesis	1789728009.000	5	357945601.900	2.981	0.056
	Error	1440972222.000	12	120081018.500[a]		
Culture Replicate(Ge Exposure Treatment)	Hypothesis	1440972222.000	12	120081018.500	18.610	0.000
	Error	232291666.700	36	6452546.296[b]		

[a] MS(Culture Replicate(Ge Exposure Treatment))
[b] MS(Error)

8 Day Comparison for Figure 3a

Tests of Between-Subjects Effects

Dependent Variable: 8 Day Cell Concentration

Source		Type III Sum of Squares	df	Mean Square	F	*p*
Intercept	Hypothesis	8406278935.000	1	8406278935.000	21.028	0.001
	Error	4797222222.000	12	399768518.500[a]		
Ge Exposure Treatment	Hypothesis	11049103010.000	5	2209820602.000	5.528	**0.007**
	Error	4797222222.000	12	399768518.500[a]		
Culture Replicate(Ge Exposure Treatment)	Hypothesis	4797222222.000	12	399768518.500	10.310	0.000
	Error	1395833333.000	36	38773148.150[b]		

[a] MS(Culture Replicate(Ge Exposure Treatment))
[b] MS(Error)

***p*-values from Tukey's HSD *post-hoc* test**	**-Ge Control**	**+1%Ge**	**+2%Ge**	**+5% Ge**	**+10%Ge**
**-Ge Control**					
**+1%Ge**	<0.001				
**+2%Ge**	<0.001	0.007			
**+5% Ge**	<0.001	<0.001	0.135		
**+10%Ge**	<0.001	<0.001	0.021	0.965	
**+20%Ge**	<0.001	<0.001	0.027	0.98	1

10 Day Comparison for Figure 3a

Tests of Between-Subjects Effects

Dependent Variable: 10 Day Cell Concentration

Source		Type III Sum of Squares	df	Mean Square	F	*p*
Intercept	Hypothesis	21650028940.000	1	21650028940.000	21.651	0.001
	Error	11999652780.000	12	999971064.800[a]		
Ge Exposure Treatment	Hypothesis	44226880790.000	5	8845376157.000	8.846	**0.001**
	Error	11999652780.000	12	999971064.800[a]		
Culture Replicate(Ge Exposure Treatment)	Hypothesis	11999652780.000	12	999971064.800	36.225	0.000
	Error	993750000.000	36	27604166.670[b]		

[a] MS(Culture Replicate(Ge Exposure Treatment))
[b] MS(Error)

***p*-values from Tukey's HSD *post-hoc* test**	**-Ge Control**	**+1%Ge**	**+2%Ge**	**+5% Ge**	**+10%Ge**
**-Ge Control**					
**+1%Ge**	<0.001				
**+2%Ge**	<0.001	<0.001			
**+5% Ge**	<0.001	<0.001	0.042		
**+10%Ge**	<0.001	<0.001	0.016	0.999	
**+20%Ge**	<0.001	<0.001	0.012	0.996	1

(b)

2 Day Comparison for Supplementary Figure 1

Tests of Between-Subjects Effects

Dependent Variable: 2 Day Cell Concentration

Source		Type III Sum of Squares	df	Mean Square	F	*p*
Intercept	Hypothesis	10153125000.000	1	10153125000.000	55.172	0.002
	Error	736111111.100	4	184027777.800[a]		
Ge Exposure Treatment	Hypothesis	125347222.200	1	125347222.200	0.681	0.456
	Error	736111111.100	4	184027777.800[a]		
Culture Replicate(Ge Exposure Treatment)	Hypothesis	736111111.100	4	184027777.800	2.064	0.149
	Error	1069791667.000	12	89149305.560[b]		
[a] MS(Culture Replicate(Ge Exposure Treatment))		10153125000.000	1	10153125000.000	55.172	0.002
[b] MS(Error)						

4 Day Comparison for Supplementary Figure 1

Tests of Between-Subjects Effects

Dependent Variable: 4 Day Cell Concentration

Source		Type III Sum of Squares	df	Mean Square	F	*p*
Intercept	Hypothesis	80500781250.000	1	80500781250.000	17.180	0.014
	Error	18743402780.000	4	4685850694.000[a]		
Ge Exposure Treatment	Hypothesis	4253472.222	1	4253472.222	0.001	0.977
	Error	18743402780.000	4	4685850694.000[a]		
Culture Replicate(Ge Exposure Treatment)	Hypothesis	18743402780.000	4	4685850694.000	13.067	0.000
	Error	4303125000.000	12	358593750.000[b]		
[a] MS(Culture Replicate(Ge Exposure Treatment))		80500781250.000	1	80500781250.000	17.180	0.014
[b] MS(Error)						

6 Day Comparison for Supplementary Figure 1

Tests of Between-Subjects Effects

Dependent Variable: 6 Day Cell Concentration

Source		Type III Sum of Squares	df	Mean Square	F	*p*
Intercept	Hypothesis	96616753470.000	1	96616753470.000	16.748	0.015
	Error	23075694440.000	4	5768923611.000[a]		
Ge Exposure Treatment	Hypothesis	94531250.000	1	94531250.000	0.016	0.904
	Error	23075694440.000	4	5768923611.000[a]		
Culture Replicate(Ge Exposure Treatment)	Hypothesis	23075694440.000	4	5768923611.000	7.834	0.002
	Error	8836458333.000	12	736371527.800[b]		
[a] MS(Culture Replicate(Ge Exposure Treatment))		96616753470.000	1	96616753470.000	16.748	0.015
[b] MS(Error)						

(c)

2 Day Comparison for Figure 3b

Tests of Between-Subjects Effects

Dependent Variable: 2 Day Cell Concentration

**Source**		**Type III Sum of Squares**	**df**	**Mean Square**	**F**	***p***
Intercept	Hypothesis	11158593750.000	1	11158593750.000	31.264	0.000
	Error	4282986111.000	12	356915509.300[a]		
Ge Exposure Treatment	Hypothesis	2248524306.000	5	449704861.100	1.260	0.342
	Error	4282986111.000	12	356915509.300[a]		
Culture Replicate(Ge Exposure Treatment)	Hypothesis	4282986111.000	12	356915509.300	7.360	0.000
	Error	1745833333.000	36	48495370.370[b]		
[a] MS(Culture Replicate(Ge Exposure Treatment))		11158593750.000	1	11158593750.000	31.264	0.000
[b] MS(Error)						

4 Day Comparison for Figure 3b

Tests of Between-Subjects Effects

Dependent Variable: 4 Day Cell Concentration

**Source**		**Type III Sum of Squares**	**df**	**Mean Square**	**F**	***p***
Intercept	Hypothesis	15504166670.000	1	15504166670.000	12.346	0.004
	Error	15069097220.000	12	1255758102.000[a]		
Ge Exposure Treatment	Hypothesis	23250694440.000	5	4650138889.000	3.703	**0.029**
	Error	15069097220.000	12	1255758102.000[a]		
Culture Replicate(Ge Exposure Treatment)	Hypothesis	15069097220.000	12	1255758102.000	25.956	0.000
	Error	1741666667.000	36	48379629.630[b]		
[a] MS(Culture Replicate(Ge Exposure Treatment))		15504166670.000	1	15504166670.000	12.346	0.004
[b] MS(Error)						

***p*-values from Tukey's HSD *post-hoc* test**	**-Ge Control**	**+5%Ge Control**	**+5%Ge +Si 0H**	**+5%Ge +NaCl 0H**	**+5%Ge +Si 24H**
**-Ge Control**					
**+5%Ge Control**	<0.001				
**+5%Ge +Si 0H**	<0.001	0.107			
**+5%Ge +NaCl 0H**	<0.001	0.978	0.02		
**+5%Ge +Si 24H**	<0.001	0.624	0.878	0.226	
**+5%Ge +NaCl 24H**	<0.001	0.999	0.209	0.894	0.82

6 Day Comparison for Figure 3b

Tests of Between-Subjects Effects

Dependent Variable: 6 Day Cell Concentration

**Source**		**Type III Sum of Squares**	**df**	**Mean Square**	**F**	***p***
Intercept	Hypothesis	25621556710.000	1	25621556710.000	8.160	0.014
	Error	37680555560.000	12	3140046296.000[a]		
Ge Exposure Treatment	Hypothesis	54104658560.000	5	10820931710.000	3.446	**0.037**
	Error	37680555560.000	12	3140046296.000[a]		
Culture Replicate(Ge Exposure Treatment)	Hypothesis	37680555560.000	12	3140046296.000	13.737	0.000
	Error	8229166667.000	36	228587963.000[b]		

[a] MS(Culture Replicate(Ge Exposure Treatment))
[b] MS(Error)

***p*-values from Tukey's HSD *post-hoc* test**	**-Ge Control**	**+5%Ge Control**	**+5%Ge +Si 0H**	**+5%Ge +NaCl 0H**	**+5%Ge +Si 24H**
**-Ge Control**					
**+5%Ge Control**	<0.001				
**+5%Ge +Si 0H**	<0.001	0.198			
**+5%Ge +NaCl 0H**	<0.001	1	0.191		
**+5%Ge +Si 24H**	<0.001	0.507	0.99	0.495	
**+5%Ge +NaCl 24H**	<0.001	1	0.206	1	0.519

8 Day Comparison for Figure 3b

Tests of Between-Subjects Effects

Dependent Variable: 8 Day Cell Concentration

**Source**		**Type III Sum of Squares**	**df**	**Mean Square**	**F**	***p***
Intercept	Hypothesis	60000000000.000	1	60000000000.000	20.223	0.001
	Error	35603472220.000	12	2966956019.000[a]		
Ge Exposure Treatment	Hypothesis	162704861100.000	5	32540972220.000	10.968	**0.000**
	Error	35603472220.000	12	2966956019.000[a]		
Culture Replicate(Ge Exposure Treatment)	Hypothesis	35603472220.000	12	2966956019.000	27.962	0.000
	Error	3819791667.000	36	106105324.100[b]		

[a] MS(Culture Replicate(Ge Exposure Treatment))
[b] MS(Error)

***p*-values from Tukey's HSD *post-hoc* test**	**-Ge Control**	**+5%Ge Control**	**+5%Ge +Si 0H**	**+5%Ge +NaCl 0H**	**+5%Ge +Si 24H**
**-Ge Control**					
**+5%Ge Control**	<0.001				
**+5%Ge +Si 0H**	<0.001	0.001			
**+5%Ge +NaCl 0H**	<0.001	1	0.001		
**+5%Ge +Si 24H**	<0.001	0.016	0.894	0.012	
**+5%Ge +NaCl 24H**	<0.001	1	0.001	1	0.014

10 Day Comparison for Figure 3b

Tests of Between-Subjects Effects

Dependent Variable: 10 Day Cell Concentration

Source		Type III Sum of Squares	df	Mean Square	F	*p*
Intercept	Hypothesis	88614004630.000	1	88614004630.000	66.229	0.000
	Error	16055902780.000	12	1337991898.000[a]		
Ge Exposure Treatment	Hypothesis	276345717600.000	5	55269143520.000	41.308	**0.000**
	Error	16055902780.000	12	1337991898.000[a]		
Culture Replicate(Ge Exposure Treatment)	Hypothesis	16055902780.000	12	1337991898.000	10.630	0.000
	Error	4531250000.000	36	125868055.600[b]		

[a] MS(Culture Replicate(Ge Exposure Treatment))
[b] MS(Error)

p-values from Tukey's HSD *post-hoc* test	-Ge Control	+5%Ge Control	+5%Ge +Si 0H	+5%Ge +NaCl 0H	+5%Ge +Si 24H
-Ge Control					
+5%Ge Control	<0.001				
+5%Ge +Si 0H	<0.001	0.001			
+5%Ge +NaCl 0H	<0.001	1	0.002		
+5%Ge +Si 24H	<0.001	0.022	0.916	0.027	
+5%Ge +NaCl 24H	<0.001	1	0.002	1	0.024

Supplementary Table 1. Results of statistical analyses of comparisons of culture cell concentrations from Ge treatment experiments. Results were generated with a nested mixed-effects General Linear Model (GLM); Ge treatment was set as a fixed effect and Culture Replicate as a random effect nested within Ge treatment. This accounts for possible variation in the cell concentrations of the various cultures used to establish each set of replicates. The model was run through SPSS v24 (first table for each time-point comparison), followed by *post-hoc* analysis of pairwise comparisons using Tukey's HSD (second tables, where present) if a significant effect of Ge treatment was observed from the GLM. Significant (<0.05) *p*-values detected by GLM are marked in **bold red**; significant (<0.05) *p*-values from Tukey's HSD *post-hoc* tests are marked in yellow highlight. (a) Comparisons of *Stephanoeca diplocostata* cell concentration at each time point at various levels of Ge exposure, corresponding to Figure 3a. After 10 days all cultures at <5%Ge exposure are significantly different while treatments with >5%Ge all have similarly low cell concentrations. This demonstrates that increasing %Ge exposure significantly reduces cell division rates up to a toxic threshold of +5%Ge. (b) Comparisons of *Salpingoeca rosetta* cell concentration at each time point at control (-Ge) and +20%Ge exposure, corresponding to Supplementary Figure 1. No significant differences between Ge treatments were detected the nested mixed-model GLM, indicating that there is no evidence that Ge treatment affects growth rates or survival of the *S. rosetta*, a non-siliceous choanoflagellate species (c) Comparisons of *S. diplocostata* cell concentration at each time point, at various combinations of Ge, Si and NaCl treatments, corresponding to Figure 3b. After 4 days the -Ge controls have significantly higher cell concentrations than all +5%Ge treated cultures. After 10 days, +Si treated cultures have significantly higher cell concentrations compared to untreated +5%Ge cultures, with no statistical difference if Si is added after 0 or 24 hours. In contrast, +NaCl treatment produces no significant effects. This is evidence that addition of Si can partially ameliorate the effect of +Ge on loricate choanoflagellate growth rates.
