# Supplementary figures and images for: A model for the effects of germanium on silica biomineralization in choanoflagellates
Source: J R Soc Interface. 2016 Sep;13(122):20160485. doi: 10.1098/rsif.2016.0485 (PMC5046948; doi:10.1098/rsif.2016.0485)

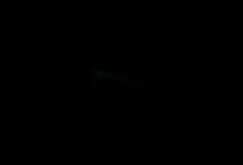

Supplement: Supplementary Stack Image 1A [file rsif20160485supp2.tif]

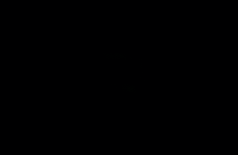

Supplement: Supplementary Stack Image 1B [file rsif20160485supp3.tif]

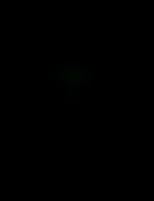

Supplement: Supplementary Stack Image 1C [file rsif20160485supp4.tif]

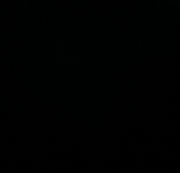

Supplement: ESM_Supplementary_Stack_Image_2 [file rsif20160485supp5.tif]
